# An Improved Chinese String Comparator for Bloom Filter Based Privacy-Preserving Record Linkage

**DOI:** 10.3390/e23081091

**Published:** 2021-08-22

**Authors:** Siqi Sun, Yining Qian, Ruoshi Zhang, Yanqi Wang, Xinran Li

**Affiliations:** Department of Mathematics and Statistics, College of Science, Huazhong Agricultural University, Wuhan 430070, China; Ssq200731@webmail.hzau.edu.cn (S.S.); zjqyn@webmail.hzau.edu.cn (Y.Q.); bjzrs@webmail.hzau.edu.cn (R.Z.); 614209595@webmail.hzau.edu.cn (Y.W.)

**Keywords:** privacy-preserving record linkage, Chinese characters, SoundShape code, Bloom filter, proportions of SoundShape code

## Abstract

With the development of information technology, it has become a popular topic to share data from multiple sources without privacy disclosure problems. Privacy-preserving record linkage (PPRL) can link the data that truly matches and does not disclose personal information. In the existing studies, the techniques of PPRL have mostly been studied based on the alphabetic language, which is much different from the Chinese language environment. In this paper, Chinese characters (identification fields in record pairs) are encoded into strings composed of letters and numbers by using the SoundShape code according to their shapes and pronunciations. Then, the SoundShape codes are encrypted by Bloom filter, and the similarity of encrypted fields is calculated by Dice similarity. In this method, the false positive rate of Bloom filter and different proportions of sound code and shape code are considered. Finally, we performed the above methods on the synthetic datasets, and compared the precision, recall, F1-score and computational time with different values of false positive rate and proportion. The results showed that our method for PPRL in Chinese language environment improved the quality of the classification results and outperformed others with a relatively low additional cost of computation.

## 1. Introduction

In the era of Big Data, it has become increasingly important to obtain more information through multisource data fusion for data analysis, and many organizations have begun to collect and process data from multiple sources to capture valuable information. The achievement of the above work usually requires linking records belonging to the same entity from multiple databases. Records can easily be linked if the unique identifiers (UIDs) of individuals are available. However, when UIDs between different databases are missing, records in these databases can be integrated and linked through probabilistic record linkage using personal identification fields (e.g., name and address) [1]. However, if we directly compare the identity information, it may lead to privacy disclosure problems. Privacy-preserving record linkage [2] (referred to PPRL) can solve the above problems well, which ensures that only the final matched record information is shared between data sources, and does not reveal the information of other unmatched records.

Researchers have proposed many methods for PPRL [3,4,5,6,7,8,9], one of which is to encrypt the fields based on Bloom filter [10,11,12] and calculate their similarity. To compare two strings, we can add a null letter on both sides of each string and split them into bi-gram [13], then store them in the Bloom filters and their similarity can be calculated by Dice coefficient.

Most of the existing PPRL algorithms were designed for alphabetic language-based datasets. However, different from alphabetic languages, Chinese are ideographic characters [14]. In Chinese, many characters are similar in pronunciation but different in shape, or similar in shape but different in pronunciation. Obviously, simply applying the existing PPRL methods for Chinese environment cannot achieve satisfactory results.

In this paper, the existing Chinese encoding methods and PPRL methods are studied, and an improved similarity calculation method based on Bloom filter is proposed to adapt the task of PPRL in Chinese environment.

Contributions: An improved calculation method based on SoundShape code was proposed to support the task of PPRL in Chinese environment. We assigned different proportions of sound codes and shape codes to study what proportion can achieve a better linkage decision. Then we conducted a comprehensive evaluation of our method using synthetic datasets and compared the accuracy of record linkage results with different proportions of sound codes and shape codes, and confirmed the outperformance of our proposed method.

Outline: The remaining part of this paper is performed as follows: we present related work about Secure computational encoding and Chinese encoding methods in Section 2. In Section 3, we describe the methods of encryption and calculating the similarity of Chinese strings and the probabilistic record linkage method proposed by Winkler (PRL-W). In Section 4, the process of adding different types of errors into synthetic datasets is described. Then different proportions are evaluated by (A) the performance measures of precision, recall, and F1-score and (B) their computational time. Section 5 is the conclusion of this paper.

## 2. Related Work

Currently, the main encoding techniques commonly applied to record linkage can be categorized into Secure Multiparty Computation (SMPC) and perturbation techniques.

Techniques based on SMPC usually employ cryptography operations, which are computationally expensive and cannot be extended to large databases. However, techniques based on perturbation can provide acceptable linkage quality and performance with adequate privacy preserving.

At present, a popular encoding technology based on perturbation technique is Bloom filter [15], which uses the q-gram for approximate matching, and calculates the similarity of the 2 Bloom filters based on the set similarity. Niedermeyer et al. [16] conduct a full cryptanalysis of the fundamental construction principle of basic Bloom filters as used in record linkage. They describe the encrypting procedure and the deciphering process in detail, coming to the conclusion that the independent hash functions are better than the double hashing scheme when being used in Bloom filters.

In terms of Chinese character encoding, Zhang [17] proposed a Chinese character coding scheme using stroke order, all encoded Chinese characters are represented by four letters. With this method, there are sometimes different characters are encoded the same way. Chen et al. [18,19,20] has proposed the input method of Chinese character phonetic-form code. Du [21] invented the holo-information code for Chinese characters by using the order of radicals and strokes of Chinese characters. The radicals in Chinese characters are classified and combined according to the order of the initial letters in the phonetic alphabet or the number of strokes.

For record linkage, it’s better to consider both shape and pronunciation when encoding Chinese characters. Wang et al. [22] proposed a method to convert Chinese characters into SoundShape code. Xu et al. [14] proposed methods for calculating the similarity of Chinese characters based on the SoundShape code to perform record linkage in Chinese environment. Their research focuses more on different methods for calculating the field similarity, which does not emphasize the privacy-preserving.

## 3. Methods

In this section, we first introduce an encoding method, Bloom filter. Then the Dice similarity and SoundShape code are discussed before we propose an improved Chinese characters encoding method. Finally, the record linkage method based on EM algorithm is described.

### 3.1. Bloom Filter

Bloom filter [23] can be used to retrieve whether an element is in a dataset. In this paper, we use Bloom filter to encrypt the identification fields of records to determine whether the two records belong to the same individual.

The Bloom filter consists of a long bit array and a series of random mapping functions (hash functions). First we set the bit array filled with zeros. Then divide the strings to be encrypted into bi-gram (adding a null character before the first letter and after the last letter). For example, the word “FILTER” becomes “_FILTER_”, and then is divided into “_F”, “FI”, “IL”, “LT”, “TE”, “ER”, “R_”. Next, each bi-gram is mapped into the previously set of bit array.

**Figure 1:**
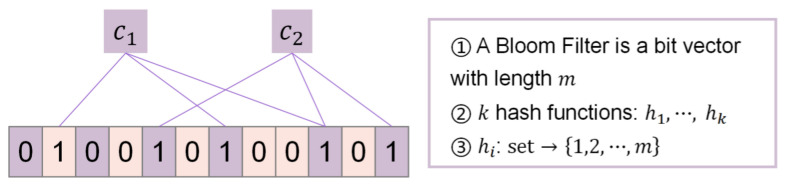
Description of Bloom filter.

A hash function can transform input data of any size to output with a fixed length hash value and the same bi-gram will produce the same hash encoding. Each hash code corresponds to a position in the bit array, the value of the this position will be set from 0 to 1 after mapping (Figure 1). Besides, there can be multiple hash functions in the Bloom filter, which means there will be multiple positions set to 1 for each bi-gram. After all the bi-gram segmented by the encrypted object are mapped by the hash function, the Bloom filter encryption is completed.

Using the Bloom filter to judge whether a pair of characters in a string are the same, just put the bi-gram through the Bloom filter, and test corresponds to an array of the number of location and position 1 are consistent. If it is consistent, it is considered that there may be dual characters in the string, but there is another possibility, that is, “false positive”, in which different characters produce hash conflicts through the hash function, resulting in an error in the result.

The estimation of false positive rate is as follows: *k* is the number of hash functions, *n* is the length of set $S=\{x_{1},x_{2},\ldots ,x_{n}\}$ and *m* is the length of the vector. To map *S* completely into the array, we need to do kn hashes. Assume that kn<m and the hash functions are completely random. The probability *p* that one bit of the bit array is still 0 is:

(1) $$p={\left(1-\frac{1}{m}\right)}^{kn}\approx e^{-\frac{kn}{m}},$$

where $\frac{1}{m}$ is the probability that any hash function picks this digit, $\left(1-\frac{1}{m}\right)$ is the probability that the hash function does not pick this digit. The fact that some digit is still 0 means that kn has not been hashed all the time, thus the probability is

(2) $${\left(1-\frac{1}{m}\right)}^{kn}.$$

In order to simplify the operation, make

(3) $$p\approx e^{-\frac{kn}{m}},$$

because $\lim_{x\to \infty }{\left(1-\frac{1}{x}\right)}^{-x}=e$. If ρ is the ratio of 0 in the array of bits, the mathematical expectation is

(4) E(ρ)=p.

Therefore, the false positive rate is:

(5) $$f={\left(1-\rho \right)}^{k}\approx {\left(1-p\right)}^{k},$$

where (1−ρ) is the ratio of 1 in a bit array, and ${\left(1-\rho \right)}^{k}$ represents the position of 1 selected for *k* hashes, which is a false positive rate. *p* is only the mathematical expectation of ρ, and in reality the value of ρ may deviate from its mathematical expectation. M. Mitzenmacher [24] has shown that the ratio of 0 in a bit array is very concentrated near its mathematical expected value. Therefore, substitute *p* into the above equation, respectively, and get:

(6) $$f\approx {\left(1-e^{-\frac{kn}{m}}\right)}^{k}.$$

If given the value of *f* as expected, the following relationship can be concluded:

(7) $$m\approx -\frac{k}{ln(1-f^{\frac{1}{k}})}n.$$

The false positive probability is minimized when satisfying

(8) $$k=\frac{m}{n}ln2$$

and

(9) $$f={\left(\frac{1}{2}\right)}^{k}.$$

Obviously, the false positive rate can be set before the experiment, which designs the optimal number of hash functions and the most appropriate length of bit array. Additionally, it also has a significant impact on the final result of the record linkage in our method, which will be discussed later in this paper.

### 3.2. Dice Coefficient

To compute the similarity between the two Bloom filters A and B, the Dice coefficient, whose value is between 0 and 1, is calculated as the sum of the number of positions with the value of 1 for both Bloom filters divided by the number of positions with the value of 1 for each of the two filters:

(10) $$Dice\left(A,B\right)=\frac{2\times Common1bits}{1bitsA+1bitsB}.$$

**Figure 2:**
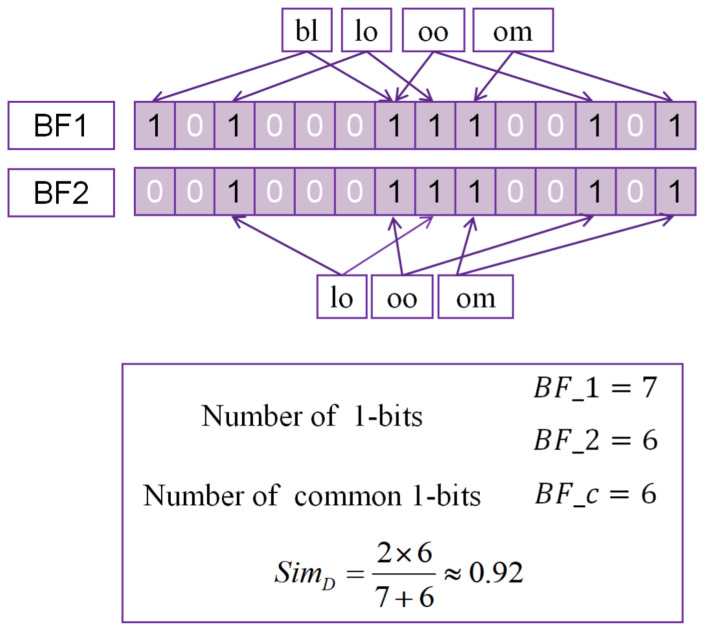
Example of Dice coefficient.

Obviously, the greater the value of Dice coefficient, the greater the similarity is (Figure 2).

### 3.3. SoundShape Code

**Figure 3:**
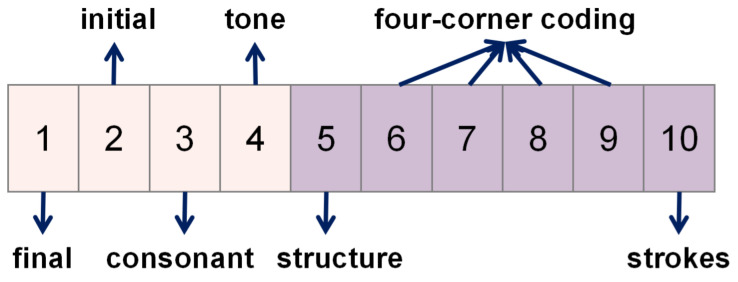
The composition of SoundShape code.

The main idea of SoundShape Code [25] (referred to as SSC) is encoding Chinese characters into strings according to the pronunciation and shape. The fixed length of the SoundShape code is 10, the first four positions constitute the sound code, while the last six positions constitute the shape code (Figure 3). The sound code part of SSC consists of initials, consonants, finals and tones of Pinyin. The shape part of SSC consists of structure, four-corner coding, and the number of a stroke to construct a Chinese character.

The first is the final of a Chinese character. Through the substitution rules in Table 1, the final of a Chinese character is transformed and placed at the first place of the SoundShape code. There are altogether 24 finals in Chinese pinyin.

The second part is the initial. Similar to the transformation of finals above, we use the following substitution rules (Table 2) to convert the corresponding part of the consonant as the second part of the SoundShape code.

The third is the consonant. Consonant is used when there is a consonant between an initial and a final, which is consistent with the mapping rules of the final table.

The fourth is the tone, where the four tones in Chinese characters are replaced by 1, 2, 3 and 4.

**Figure 4:**
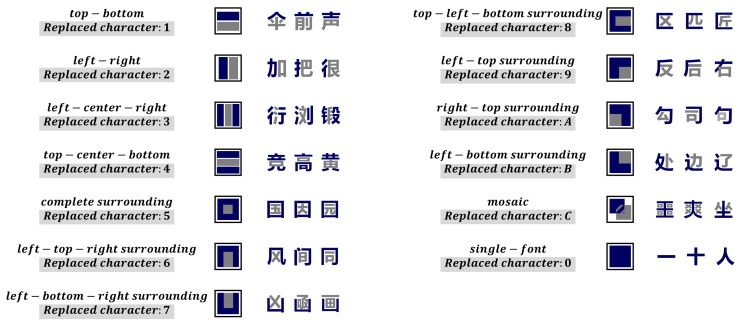
The structure of the Chinese character.

The fifth is the structure. Chinese characters can be divided into simple characters and compound characters while there are twelve classifications in compound characters. Replacing the structure of the Chinese character with the characters in Figure 4, and placing it in the SoundShape code.

The sixth to ninth positions are four-corner coding, which are encoded in the order of upper left, upper right, lower left and lower right corners.

The tenth code is the number of strokes of a Chinese character. For characters with a number of strokes from one to nine, the numbers 1–9 are used. It is stipulated that ’A’ corresponds to 10 strokes, ’B’ to 11 strokes, and so on, until ’Z’ is used for the number of strokes greater than or equal to 35.

Based on the above description, it can be obtained that the SoundShape codes of “中”, “国”, “北” and “京” (“中国” meaning “China”, “北京” meaning “Beijing”) is, respectively, “KE01C50004”, “2852560108”, “7103112115” and “HB01400908”.

After converting each character into a SoundShape code, we add them and take modulus 35 by experience. For example, as shown in Table 3, the initial of the SoundShape code for “中”, “国”, “北” and “京” are “K”, “2”, “7” and “H”, respectively. “K” is the number 20 and “H” is the number 17, so the combined income is 46. Furthermore, then we take modulus 35, the final result is B, which is representing number 11.

However, strings composed with the same characters but in different order produce the same SoundShape code. For example, “中国北京” share the same SoundShape code with “京北国中”. To correct this defect, each character is multiplied by its position number and then add it them and take modulo 35. For example, the word “中” in “中国北京” is the first in the sequence and so on, the initial digit of the SoundShape code for “中”, “国”, “北” and “京” are “K”, “2”, “7” and “H”, respectively. Therefore, as shown in Table 4, $x_{1}=``$K”$\times 1=20,x_{2}=2\times 2=4,x_{3}=7\times 3=21,x_{4}=``$H’’×4=68, then add them up to 113, and the result becomes 8 after modulus 35. Therefore, the final shape code of “中国北京” is “87AI6K663W” (Table 5) .

### 3.4. Proposed Method for Similarity Calculation

According to the encoding method of SoundShape Code (SSC), we can convert Chinese characters into the corresponding 10-digit code composed of letters and numbers. The similarity of two records can be obtained by comparing their corresponding SSC.

For the two Chinese characters A and B, we first convert them to SSC, $SSC_{A}=\left[a_{1},a_{2},a_{3},...,a_{10}\right]$ and $SSC_{B}=\left[b_{1},b_{2},b_{3},...,b_{10}\right]$. Therefore, $SSC_{A}$ and $SSC_{B}$ can obtain their Dice similarity through Equation (10).

Table 6 shows an example of four Chinese names and their corresponding SSCs. Among them, the SSC of “晨” (chen) and “辰” (chen) are similar in the sound code part, while the SSCs of “住” (zhu) and “往” (wang) are similar in the shape code part.

However, the above-mentioned method cannot handle the privacy disclosure problems, so we propose an improved similarity calculation method based on Bloom filter. First, we encrypt the first four sound codes and the last six shape codes with Bloom filter, respectively,

(11) $$\left\{\begin{array}{c} bfsound_{A}=BF\left(a_{1},a_{2},a_{3},a_{4}\right)\\ bfshape_{A}=BF\left(a_{5},a_{6},a_{7},a_{8},a_{9},a_{10}\right) \end{array}\right.$$

(12) $$\left\{\begin{array}{c} bfsound_{B}=BF\left(b_{1},b_{2},b_{3},b_{4}\right)\\ bfshape_{B}=BF\left(b_{5},b_{6},b_{7},b_{8},b_{9},b_{10}\right) \end{array}\right.$$

then the following equation can be used to calculate the similarity between A and B:

(13) $$dice\left(A,B\right)=\alpha Dice\left(bfsound_{A},bfsound_{B}\right)+\left(1-\alpha \right)Dice\left(bfshape_{A},bfshape_{B}\right),$$

where α is the proportion of sound code and shape code which is based on the rule of thumb. Table 7 shows the similarity of the same field pair as in Table 6 with α=0.5. In our work, the final result of the record linkage is different with the different value of α.

### 3.5. Probabilistic Record Linkage Proposed by Winkler

Probabilistic record linkage proposed by Winkler [26] (PRL-W) is an extension of Fellegi and Sunter [27] approach (PRL-FS). Taking into account approximate matches, the PRL-W method decomposes the string comparator values into different and non-intersecting sub-intervals of [0,1]. The m-probability $m_{i,s}$ is the conditional probability that the similarity of two records belonging to the same entity in the field *i* falls in the interval *s*, and the u-probability $u_{i,s}$ is the conditional probability that the similarity of the field *i* of two records belonging to different entities falls in the interval *s*. These results can be achieved by manually evaluating the quality of the comparison or by comparing it with the current database. This paper uses the EM algorithm [28] to estimate the values of these two probabilities. Then, depending on the $m_{i,s}$ and $u_{i,s}$, the weight of the similarity of field *i* in the interval *s* is calculated for:

(14) $$w_{i,s}=\log_{2}\left(\frac{m_{i,s}}{u_{i,s}}\right)$$

and the weight of the two records are calculated by adding all field’s weight:

(15) $$\sum \left(w_{i,s}\times I\left[\gamma_{i}\in S\right]\right),$$

where $\gamma_{i}$ is the similarity of two records in field i.

Finally, a linkage decision rule can be found using the option value of the parameter (the proportion of the pair of records associated with the same entity):

If $w\geq T_{C}$, the record pair is considered as match.

If $w<T_{C}$, the record pair is considered as non-match.

Where the threshold $T_{C}$ is the *p*th quantile of the weights of all record pairs in descending order.

## 4. Experiments

### 4.1. Dataset Construction

**Figure 5:**
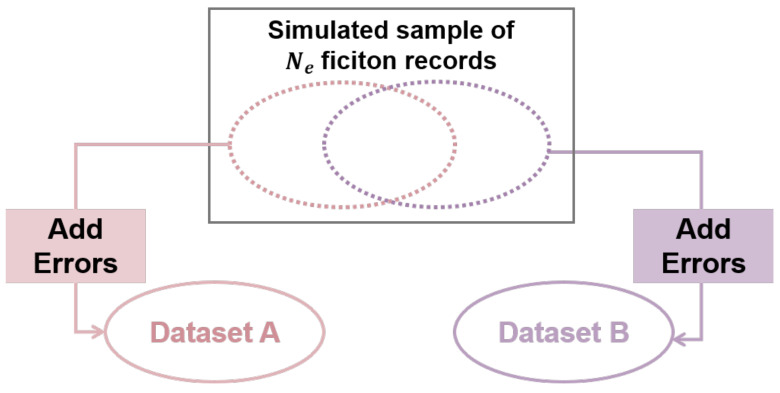
The process of generating synthetic datasets.

In order to evaluate the effectiveness of PPRL, we conducted our study on the synthetic datasets. In addition, we need to control the quality of the synthetic dataset (error ratio and type per dataset) to ensure that the synthetic dataset is effective in assessing the performance of the record linkage method (true matches, type and error rate). As shown in Figure 5, we generated two datasets containing real-world noise using the following methods:

Step 1: Generate the Dataset

The datasets A and B are generated by random sampling (without replacement) from $N_{e}$ fictional records. In this study, we set $N_{e}=1500$ and $N_{A}=N_{B}=888$. The value of the overlap rate (the percentage of the true matches), γ is randomly generated to simulate the most realistic environment (Table 8). Each record in the synthetic dataset contains four fields: name, address, sex, date of birth. Besides these, there is also a unique identification key for determining whether a record pair corresponds to the same entity. Here is an example of one of these records.

<Name> 蔡宇 (Cai Yu) <Name>
<Address> 山西省运城地区永济市 (Yongji, Yuncheng, Shanxi) <Address>
<Sex> 女 (female) <Sex>
<Birthdate> 19970621 <Birthdate>
<ID> 2019308110080 <ID>

Step 2: Add Errors

A random selection of records in datasets A and B is followed by an error ratio (no error is introduced in the identification key). We studied on Chinese handwritten forms recognized by OCR and and found that the accuracy of each identification field of handwritten recognition was around 70%. With this specification, the errors are added randomly to datasets A and B: the proportion of records with no errors to the total data is in the interval [0.7, 0.73], the proportion of records with one error to the total data is in the interval [0.2, 0.23], and the proportion of records with two errors to the total data is in the interval [0.02, 0.04].

1. 1. Substitution
  In the dataset, substitution errors often occur due to handwriting errors, scanning errors, oral transmission and other problems. We divide the error into the phonetic error and spelling error. For example: (1) characters with the same pronunciation but different shapes: “张” (zhang) and “章” (zhang); (2) Similar shapes: “闪” (shan) and “问” (wen). We use the Chinese homophone lexicon and Chinese type near lexicon to randomly replace the characters in dataset B.

**Example 1**. *Replace “宇” (yu) by “雨” (yu) in Step 1 because the two words have the same pronunciation.*

<Name> 蔡雨 (Cai Yu) <Name>
<Address> 山西省运城地区永济市 (Yongji, Yuncheng, Shanxi) <Address>
<Sex> 女 (female) <Sex>
<Birthdate> 19970621 <Birthdate>
<ID> 2019308110080 <ID>

**Example 2**. *Replace “宇” (yu) by “宁” (ning) because the two characters have the similar shape.*

<Name> 蔡宁 (Cai Ning) <Name>
<Address> 山西省运城地区永济市 (Yongji, Yuncheng, Shanxi) <Address>
<Sex> 女 (female) <Sex>
<Birthdate> 19970621 <Birthdate>
<ID> 2019308110080 <ID>

2. Denormalization
  It is heterogeneous for information in real datasets because different organizations have their own requirements for data quality. Thus, the data collected, such as addresses may not be uniform. As a result, we produce some nonuniform information in the synthetic dataset. For example, we randomly delete some information in the address field (such as province, city or region), and the city in the address in data is omitted:
  <Name> 蔡宇 (Cai Yu) <Name>
  <Address> 山西省运城地区 (Yuncheng, Shanxi) <Address>
  <Sex> 女 (female) <Sex>
  <Birthdate> 19970621 <Birthdate>
  <ID> 2019308110080 <ID>

**Table 8:** The value of overlap rate γ in different experiments.

Experiment Number	The Value of γ	Experiment Number	The Value of γ
1	0.72	6	0.57
2	0.74	7	0.29
3	0.56	8	0.52
4	0.56	9	0.74
5	1.00	10	0.42

### 4.2. Experimental Design

We generated 888 record pairs. Firstly, 888 record pairs were encoded into the SoundShape code, and were encrypted by Bloom filter. The PPRL in Chinese environment was performed with Python 3.7 using a computer with a CPU Quad-Core Intel Core i7 2.9 GHz and 16 GB RAM.

In the experiment, we use SoundShape code to encode the field “Name”, “Address” and “Sex” separately. During the encoding by Bloom filter, it is done on the field level with the same Bloom filter parameters. The false positive rate of the Bloom filter varied from 0.1 to 0.6 and different proportions of sound codes and shape codes are used, such as (1) 50% sound codes and 50% shape codes, (2) 40% sound codes and 60% shape codes, and (3) 30% sound codes and 70% shape codes, so as to calculate the Dice similarity of the dataset. PRL-W was used to conduct interval classification of the similarity. The similarity of the data pairs was processed with latent variables, and the EM algorithm was used to estimate the parameters and make linkage between the record pairs with higher weight.

We studied the influence of different false positive rate of Bloom Filter and proportion of similarity calculation in Equation (10) by using the measures of precision, recall, F1-score [29]. The validity of the proposed method was proved by the above assessment. Finally, the running time of different methods was also compared.

## 5. Results

Through experiments, the mean value and variance of F1-score at different false positive rate and different proportions are calculated. It can be observed from Table 9 that when the α equals to 0.4, 0.5 and 0.6, the variance of F1-score of each false positive rate is relatively small, which means the results are more stable. In addition, it can be seen in the table that the F1-scores obtained by the the original SSC similarity are the minimum regardless of the value of the false positive rate. This results can show the advantages of our proposed method. The F1-score of SSC similarity is basically the minimum in each false positive rate , reflecting the advantages of the proposed method. When the false positive rate of Bloom filter is 0.3, the mean value of F1-score is generally higher.

**Figure 6:**
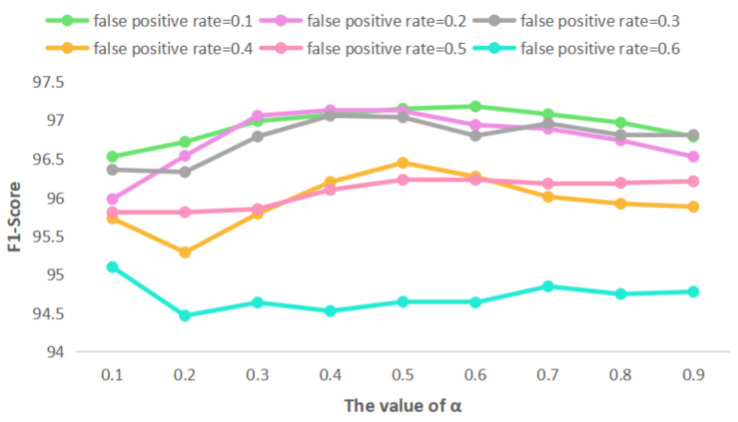
F1-score of classification by using different false positive rate and the value of α.

In order to see the comparison of results more intuitively, the following line chart is drawn. We present the F1-score of classification based on the improved similarity calculation method by setting different proportions from 0.1 to 0.9 and different false positive rates from 0.1 to 0.6. In the experiment, it can be clearly observed that the broken line with the false positive rate of 0.6 is at the bottom of Figure 5, which means it has the lowest F1-score. The result of false positive rate = 0.1, 0.2 and 0.3 are basically similar when the value of α equals 0.4 to 0.5. When the false positive rate = 0.1 and the value of α belongs to 0.4 to 0.6, the F1-score of the classification results are basically the same and better than other points (Figure 6).

While using the optimal parameter false positive rate = 0.1 and different α = 0.4, 0.5 and 0.6, we compared the proposed method with existing method (directly converting characters to SoundShape code), then use PPRL based on Bloom filter (referred to SSC similarity). From Table 10, SSC similarity performed worst in precision, recall, and F1-score. While for α = 0.6, the result performed slightly better than the the result with α = 0.4 and 0.5 in these three performances. Overall, the proposed method that combines information from SSCs and Dice similarity performs best in record linkage. Although the three performances were best when α = 0.6 among α = 0.4, 0.5 and 0.6, the difference between the three is not that obvious.

We summarized the results of with the 10 paired data sets, and counted the computational time of the different methods. Through practice, it has been proved that the accuracy of the encryption from sound code and shape code, respectively, is higher than that of the whole encryption from SoundShape code. The calculation time of different parameters in the improved method is different. First of all, the running time of the improved method is longer than that of SSC similarity, especially when α = 0.5 and 0.6. While α = 0.6, there has a slight increase in running time than α=0.5. However, taking quality and efficiency together, when α = 0.6, the benefit of quality improvement is far greater than the slight increase in time. Compared with the original methods of SoundShape code for PPRL based on Bloom filter, our method can improve the quality of the classification results on the basis of only 0.296 s increase in time (Table 11).

## 6. Conclusions

In data sharing, privacy protection has become an inevitable concern. At present, most of the existing PPRL algorithm are designed and applied for alphabetic language-based datasets. Therefore, in this paper, an improved similarity calculation method based on SoundShape code is proposed to adapt the task of PPRL in the Chinese environment. The Chinese characters are encoded into SoundShape code and are encrypted by Bloom filter. Then the similarity of encrypted fields is calculated by Dice similarity and PRL-W is used to finish the work. We compared the different proportions of sound code and shape code, and discuss the result with different false positive rates of the Bloom filter on synthetic data sets. Our proposed method shows its outperformance in precision, recall and F1-Score.

However, there are some deficiencies with our algorithm in certain cases. The information required by different users may be diversified, the data types used in this experiment are not extensive enough. Moreover, Our method mainly focuses on character similarity matching (based on SoundShape code) without considering semantic matching. In addition, the algorithm should be test on a real dataset for a better realistic evaluation and the prove of practicality. Therefore, in the future, we will collect more data types, experimenting and testing our algorithms in more realistic and complex datasets. Moreover, methods such as NER (Named Entity Recognition) will be introduced into the work for field address, so that the proposed method could be used in a wider range of applications.

## Figures and Tables

**Table 1:** 24 Finals in Chinese pinyin.

FINAL											
a	–	1	o	–	2	e	–	3	i	–	4
u	–	5	v	–	6	ai	–	7	ei	–	7
ui	–	8	ao	–	9	ou	–	A	iu	–	B
ie	–	C	ve	–	D	er	–	E	an	–	F
en	–	G	in	–	H	un	–	I	ven	–	J
ang	–	F	eng	–	I	ing	–	H	ong	–	K

**Table 2:** 23 Initials in Chinese pinyin.

INITIAL											
b	–	1	p	–	2	m	–	3	f	–	4
d	–	5	t	–	6	n	–	7	l	–	7
g	–	8	k	–	9	h	–	A	j	–	B
q	–	C	x	–	D	zh	–	E	ch	–	F
sh	–	G	r	–	H	z	–	E	c	–	F
s	–	G	y	–	I	w	–	J			

**Table 3:** Structure of the Chinese character.

Character	SoundShape Code									
中	K	E	0	1	C	5	0	0	0	4
国	2	8	5	2	5	6	0	1	0	8
北	7	1	0	3	1	1	2	1	1	5
京	H	B	0	1	4	0	0	9	0	8
Sum	B	Y	5	7	M	C	2	B	1	P

**Table 4:** Structure of the Chinese character.

Character	Digit	Position	Calculation	Result
中	K	1	“*K*”×1	20
国	2	2	2×2	4
北	7	3	7×3	21
京	H	4	“*H*”×4	68
Sum				113

**Table 5:** Replacement for the structure of the Chinese character.

Character	SoundShape Code									
中	K	E	0	1	C	5	0	0	0	4
国	2	8	5	2	5	6	0	1	0	8
北	7	1	0	3	1	1	2	1	1	5
京	H	B	0	1	4	0	0	9	0	8
Sum	8	7	A	I	6	K	6	6	3	W

**Table 6:** An example of calculating the SSC similarity.

Character	SSC	Dice Similarity
陈欣晨(Chen Xinchen)	EV03WK06YL	0.736
陈欣辰(Chen Xinchen)	EV034L16YH	
李住(Li Zhu)	9S0GBQ0Y17	0.526
李往(Li Wang)	JX5FBQ0Y18	

**Table 7:** An example of calculating SSC similarity with α=0.5.

Character	SSC	Dice Similarity of Sound Codes	Dice Similarity of Shape Codes	Results Similarity
陈欣晨 (Chen Xinchen)	EV03WK06YL	1.000	0.200	0.600
陈欣辰 (Chen Xinchen)	EV034L16YH			
李住 (Li Zhu)	9S0GBQ0Y17	0.000	0.857	0.428
李往 (Li Wang)	JX5FBQ0Y18			

**Table 9:** The mean value and variance of F1-score at different false positive rate and proportions.

	Rate = 0.1	Rate = 0.2	Rate = 0.3	Rate = 0.4	Rate = 0.5	Rate = 0.6
the original SSC similarity	93.52 ± 2.33	93.58 ± 2.45	94.37 ± 2.54	94.79 ± 2.38	94.06 ± 2.12	94.81 ± 2.73
α=0.1	96.53 ± 1.39	95.98 ± 1.01	96.36 ± 1.29	95.73 ± 1.39	95.81 ± 1.73	95.10 ± 1.51
α=0.2	96.72 ± 1.28	96.54 ± 1.32	96.33 ± 1.09	95.29 ± 1.08	95.81 ± 1.73	94.47 ± 1.48
α=0.3	96.99 ± 1.35	97.06 ± 1.34	96.79 ± 1.19	95.79 ± 0.97	95.85 ± 1.22	94.64 ± 1.48
α=0.4	97.07 ± 1.39	97.13 ± 1.37	97.06 ± 1.24	96.20 ± 0.91	96.10 ± 1.06	94.53 ± 0.62
α=0.5	97.15 ± 1.42	97.12 ± 1.33	97.04 ± 1.23	96.45 ± 0.97	96.23 ± 0.94	94.65 ± 0.55
α=0.6	97.18 ± 1.44	96.94 ± 1.35	96.80 ± 1.09	96.27 ± 0.83	96.23 ± 0.93	94.64 ± 0.51
α=0.7	97.08 ± 1.35	96.89 ± 1.29	96.96 ± 1.19	96.01 ± 0.71	96.18 ± 0.83	94.85 ± 0.55
α=0.8	96.97 ± 1.18	96.74 ± 1.06	96.81 ± 1.12	95.92 ± 0.67	96.19 ± 0.85	94.75 ± 0.49
α=0.9	96.79 ± 1.04	96.53 ± 0.89	96.81 ± 1.11	95.88 ± 0.70	96.21 ± 0.82	94.78 ± 0.47

**Table 10:** Results of record linkage on synthetic datasets.

Method	Precision	Recall	F1-Score
the original SSC similarity	93.77	93.29	93.52
Proportion with α=0.4	97.16	96.99	97.07
Proportion with α=0.5	97.27	97.04	97.15
Proportion with α=0.6	97.32	97.04	97.18

**Table 11:** Computational time for different methods.

Method	Runtime (s)
the original SSC similarity	21.67
Proportion with α=0.4	13.98
Proportion with α=0.5	13.29
Proportion with α=0.6	13.58

## Data Availability

The data used to support the findings of this study are available from the corresponding author upon request.
